# Diseases of the Antrum of Highmore; a Study of One Hundred and Fifty Cases

**Published:** 1899-10

**Authors:** L. C. Cline

**Affiliations:** Indianapolis, Ind., Professor of Laryngology and Rhinology, Medical College of Indiana


### Diseases of the Antrum of Highmore; a Study of One Hundred and Fifty Cases.

BY L. C. CLINE, M.D., INDIANAPOLIS, IND., PROFESSOR OF LARYNGOLOGY AND RHINOLOGY, MEDICAL COLLEGE OF INDIANA.

Presented to the Section on Laryngology and Otology, at the Fiftieth Annual Meeting of the American Medical Association, held at Columbus, Ohio, June 6-9, 1899.

My apology for presenting the often discussed subject, "Diseases of the Antrum of Highmore," is the confusion among observers regarding the etiology and pathologic conditions found in these cases, as well as the discrepancy of opinions as to treatment. After an experience with 150 cases, 140 of which were in my own practice, I feel warranted in calling attention to a few points noted under my observation.

My cases have all occurred between the ages of twenty and seventy. Forty-eight occurred in females and 102 in males. The disease did not predominate with any particular class of people--doctors, lawyers, ministers, teachers and tradesmen—in fact, all the callings and conditions in life were represented. Of the 140 cases, six had sarcoma, three in women and three in men. Two of these were operated on by the late Dr. J. W. Marsee, and in one of them the entire superior maxillary bone was removed, the other only partially, which gave only temporary relief from pain. The other four when apprised of the nature of the disease, refused operation and finally succumbed to the malady. These cases all gave a history of suffering from their teeth prior to the development of the disease, which fact leads me to believe that the long-continued irritation from an abscessed root discharging into the antrum is a factor in the production of sarcoma.

Four cases of empyema of the antrum have come under my observation. The symptoms were the same as those described by Dr. D. B. Kyle in his cases—that of escaping gas from abscessed and carious teeth into the antrum, producing a sense of nasal pressure with paroxysms of a dull, heavy, sickening headache. These were all relieved by extracting or treating the diseased teeth.

The etiology of my cases could all be fairly well traced to three sources, viz: dental, nasal and la grippe. As nearly as I can estimate, 50 percent were due to diseased teeth, 40 percent to sequelæ of la grippe and teeth combined and 10 percent to ethmoiditis and the various nasal obstructions. Probably a greater percentage should be assigned to la grippe complications. My estimates have been placed on the clinical history as given, which is often misleading.

Twenty cases were acute, complicated with influenza, and all subsided without operation. Of the other 120 all were chronic, suppurative cases of from two months' to seven years' standing. Operation revealed a marked swollen edematous condition of the mucous lining of the antrum in sixteen of these. In none did I find true polypoid growths, as described in some of the textbooks. Curettement was done in six, and packing with iodoform gauze in four cases. The others yielded to hot astringent douches. Five cases were bilateral. Of the rest, 75 percent were on the right side. In trying to account for this, dentists and dealers in dental supplies tell me that a large percentage of the teeth and plates that are broken occur on the left side, which goes to show that there is more biting and chewing on the left side, thus favoring decay on the right. Ethmoiditis was observed as a complication in eleven cases, all of which were preceded by la grippe. Two cases that were carefully diagnosed would not submit to operation, and, so far as I know, they are still suffering from the disease.

The zeal of some of our dental brethren in crowning, building and maintaining bridge work, I am led to believe, is a cause of empyema, in some cases at least. The thought wassuggested in six under my observation, having had to remove diseased roots under expensive bridge work before a cure could be effected. A purulent discharge from the antrum, when due to dental origin, is carious and fetid, but when its cause is from other sources, like the ethmoid and frontal sinuses, it is creamy and almost without odor.

For diagnosis I rely principally on the use of peroxid of hydrogen and the position of the head. After cleansing the nose and cocainizing, a few drops of peroxid are injected with a small syringe armed with a canula, the point of which is bent at a right angle and carried into the ostium maxillary. If pus is present, it will be manifest by the characteristic reaction. In cases of nasal obstruction or a deflected septum, I make an exploratory puncture with a small, sharp-pointed drill, through which peroxid is injected.

Illumination is less reliable and more complicated than the above method, although it is a useful aid in determining the condition of the roots of the teeth. I have come to believe, after trying the different methods of opening the antrum, that entering through the alveolar route is by far the best, for the reasons that the after-treatment is less painful, and the drainage is more complete, and the patient can, with greater ease and facility, keep the antrum clean.

Of the 118 cases operated on, all but two had one or more carious teeth, or they had already been removed. So that the objection to opening through the alveolar process on account of the teeth was reduced to a minimum.

My experience in puncturing the antrum from the outside under the ginglymoid fold has not been flattering. In every instance I had a swollen cheek from purulent infection from the discharge. The plan now followed in operating is to first enter the antrum with a small, pointed drill run by an electric motor, then insert a bit of cotton saturated with 10 or 20 percent solution of cocain well through the hole, which soon enables me to enlarge the opening to any required size. When a tube is required to keep the hole open, or food from entering, I use one made from silver wire turned to form a shoulder on the end to prevent its entering the antrum. The tube I now show you will remain where it is placed without anchorage, and the patient can remove and replace it at will. Some cases do better without a tube, using a plug of cotton instead, frequently changing it.

The time required to cure a case depends on the size of the opening and the thoroughness of the operation in removing all the carious teeth and nasal obstructions, together with thorough cleansing, curetting, packing and stimulating to healthy granulation. My cases have varied in the time required to effect a cure, from three weeks to one year, the average being from three to six months. Many cases will relapse and require reopening, after closing, on taking cold. For this reason, tonics and attention to the general health must not be neglected.

My practice has been to have the antrum thoroughly cleansed twice a day until the discharge lessens, then once daily until the discharge ceases, first using a little peroxid in water, followed by a solution of hot boric acid, or salt water; once or twice a week a solution of silver nitrate or iodin of sufficient strength to make an impression on the mucous membrane is injected. In the boggy, swollen, edematous cases the best results were obtained by using hot water injections three times a day, with a little boric acid or salt added.

The so-called dry treatment by insufflating powders has not come up to expectations. The best results observed from the use of powders were by first washing the antrum clean, and then covering the membrane with equal parts of finely powdered boric acid and lactopeptin.

In tabulating cures, I find it difficult to keep track of all cases, as they are scattered over a large territory, some changing location. But, from my knowledge of the cases, a large percentage of them have been cured.

To summarize: The points I wish to emphasize are: 1. The great number of cases that are traceable to la grippe. 2. The absence in my cases of polypoid growths. 3. The greater predominance on the right side. 4. The importance of a good-sized opening, and the removal of all diseased teeth. 5. In my experience, to open through the alveolar process is by far the best. 6. Hot douching to relieve the edematous conditions. 7. The dry treatment alone after a first washing has not been a success in my hands.

